# Current Utilization of Gel-Based Scaffolds and Templates in Foot and Ankle Surgery—A Review

**DOI:** 10.3390/gels11050316

**Published:** 2025-04-24

**Authors:** Julia E. Ralph, Bradley J. Lauck, Charles B. Colson, Santita Ebangwese, Conor N. O’Neill, Albert T. Anastasio, Samuel B. Adams

**Affiliations:** 1 Duke University School of Medicine, Durham, NC 27710, USA; julia.ralph@duke.edu; 2 University of North Carolina School of Medicine, Chapel Hill, NC 27599, USA; bradley_lauck@med.unc.edu; 3 Georgetown University School of Medicine, Washington, DC 20007, USA; 4 Department of Orthopedic Surgery, Duke University School of Medicine, Durham, NC 27710, USA; conor.n.oneill@duke.edu (C.N.O.); samuel.adams@duke.edu (S.B.A.)

**Keywords:** gels, foot and ankle surgery, scaffolds, osteochondral lesions, arthritis, templates

## Abstract

As tissue engineering and regenerative medicine (TERM) continues to revolutionize medicine and surgery, there is also growing interest in applying these advancements to foot and ankle surgery. The purpose of this article is to provide a comprehensive review of the types of gel scaffolds and templates, their applications in foot and ankle surgery, the challenges with current utilization, and the future directions of TERM in foot and ankle surgery. With multiple compelling scaffold prospects across the numerous natural, synthetic, and hybrid polymers currently utilized in TERM, promising results have been described in the treatment of osteoarthritis (OA) and osteochondral lesions (OCLs). However, concerns with material biocompatibility, structural integrity, feasibility during surgery, and degradation still exist and limit the extent of utilization. As researchers continue to develop enhanced polymers and formulations that address current issues, there are many opportunities to increase applications across foot and ankle surgery.

## 1. Introduction

In recent years, significant advancements in tissue engineering and regenerative medicine have redefined medicine and surgery [1]. Tissue engineering and regenerative medicine (TERM) encompasses a diverse field that focuses on the utilization of biomechanical materials for the repair and healing of pathological tissues [1,2,3]. The targeted approaches of TERM include in situ tissue regeneration, implantation of patients’ cultured cells, and application of in vitro biologic or biomimetic agents from cells and scaffolds [4]. In vitro engineering of these biologics involves culturing cells on a bioactive scaffold that influences three-dimensional (3D) growth via mechanical and chemical signals [4,5]. These scaffolds allow the formation of an extracellular matrix (ECM) that mimics those that occur naturally, and the scaffolds ultimately regress once implanted into the patient [5].

Hydrogels have emerged as a popular scaffold choice due to their structure and composition [6]. With over 90% of their weight made up of water, hydrogels are polymeric biomaterials that are extremely hydrophilic and can mimic the in vivo environment [7,8,9]. Ideal scaffolds allow for the diffusion of nutrients and waste products throughout the matrix to facilitate growth and proliferation [10]. Additionally, they provide stability to the growing tissue, which in turn promotes structural responses by mature cells [11]. Porosity is also a significant consideration for these scaffolds, with certain pore sizes and densities having advantages and disadvantages. Pores facilitate cell attachment, growth, and vascularization [12]; however, porosity that is too high can lead to mechanical strength and adhesion deficits [13,14,15]. Selecting scaffolds requires careful consideration of the advantages and disadvantages of gel characteristics in order to choose the most appropriate material for the desired tissue regeneration.

Gel scaffolds have been utilized in various joints and locations throughout the body. The most common current applications include bone tissue engineering, dermal regeneration, soft tissue repair, corneal endothelial cell transplantation, and cartilage repair [2,16,17,18,19]. In the knee, scaffolds have been considered and utilized for cartilage defects and meniscus repairs [20,21,22]. Others have proposed their application in intervertebral disc regeneration in patients with spinal pathologies [6]. Looking at the foot and ankle specifically, orthopedic biologics have been investigated to treat various pathologies, including acute trauma, arthritis, osteochondral defects, and tendinopathy, showing significant promise [23].

While the development and utilization of gel scaffolds and templates in foot and ankle surgery has increased, there is a limited amount of literature reviewing the recent advancements and current uses of such biotechnology. The purpose of this review is to provide a comprehensive overview of the types of gel scaffolds and templates, their applications in foot and ankle surgery, the challenges faced with current utilization, and the future directions of TERM in foot and ankle surgery.

## 2. Types of Gel Scaffolds and Templates

Many types of gel scaffolds have been proposed and developed for foot and ankle surgery. Below is a review of the materials currently utilized across orthopedic subspecialties and those that have shown promising results for future applications.

### 2.1. Natural

Natural polymers have been widely used in gel scaffolds as they mimic the natural tissue microenvironment, facilitate adequate physical and chemical responses from cells, and do not provoke an immune response from the host upon the implantation of foreign material [24,25]. Additionally, natural polymers are widely abundant in adult mammalian tissues, making them easily accessible and inexpensive to utilize [8]. Despite this, the disadvantages of natural polymers include processing difficulties and variability between material batches [26,27]. Commonly used natural polymers for tissue engineering in foot and ankle surgery include alginate, chitosan, collagen, gelatin, and hyaluronic acid (HA), which are often used in combination with one another for enhanced function.

#### 2.1.1. Alginate

**Figure 1:**
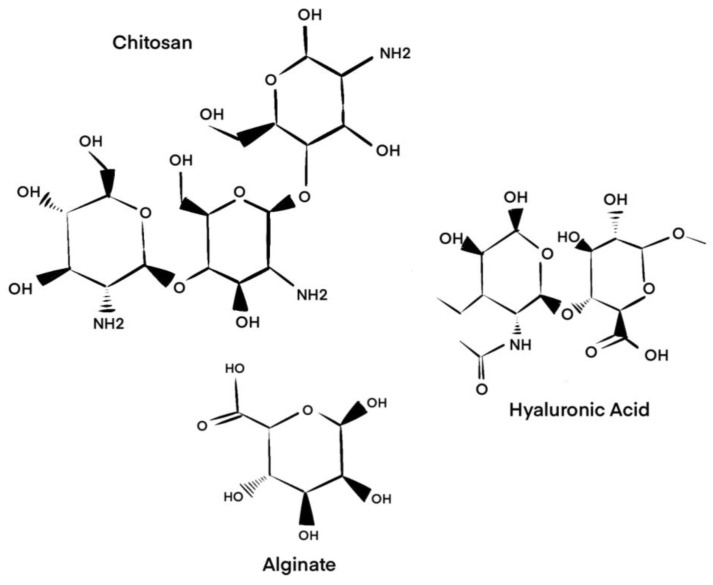
Molecular structures of alginate, chitosan, and hyaluronic acid.

Alginate is a naturally occurring polysaccharide extracted from brown seaweed and certain bacteria. After cellulose, it is the most abundant biopolymer in the world [28]. Composed of (1–4)-bonded β-d-mannuronic acid and α-l-guluronic acid, alginate has grown in popularity in the field of tissue engineering due to its strong biocompatibility, biodegradability, and low cost [28,29]. Combining sodium and divalent cations, such as Ca^2+^ and Mg^2+^, with alginate produces versatile hydrogel forms [30]. Through gas foaming, freeze-drying, solvent casting, particulate leaching, and phase separation, porous scaffolds can be created and ultimately utilized in tissue engineering processes [31]. When combined with HA, this dynamic hydrogel offers permeability for growth factors to diffuse and initiate the remodeling and healing of tissues [32] (Figure 1).

#### 2.1.2. Chitosan

Structurally similar to naturally occurring glycosaminglycans, chitosan is a linear chitin-derived polysaccharide from crustacean exoskeletons [33]. It is chemically composed of N-acetyl-d-glucosamine, which is linked to (1–4)-linked d-glucosamine via N-deacetylation [34,35]. As a result of its structure, chitosan exhibits low toxicity, high biocompatibility, strong adhesion, and preferred degradation properties [36]. To prepare chitosan in gel form, the prior literature describes techniques consisting of acidic dilution, solvent expulsion, glutalderhyde cross-linking, thermal manipulation, and UV irradiation [34,37]. Specific chitosan-based gel scaffolds used in foot and ankle surgery include BST Car-Gel (Smith and Nephew, Memphis, TN, USA) and ChitoCare (Primex, Siglufjörður, Iceland) [38,39] (Figure 1).

#### 2.1.3. Collagen

As an extremely abundant mammalian protein and a main component of natural ECMs, collagen is commonly used for many gel scaffolds and templates [40]. It expresses excellent biocompatibility and durability as a self-aggregating triple helical structure that can be easily modified to improve strength and inhibit degradation [8]. Individual collagen strands are held together by hydrogen and covalent bonds, which can in turn assemble into fibers [41]. Additionally, collagen is an extremely diverse molecule with at least 19 known distinguishable types, allowing for multiple varieties of gels with similar molecular structures and biochemical properties [40]. Collagen-based gel scaffolds can be further enhanced through chemical cross-linking modifications and combinations with other common biomedical materials, such as alginate, chitosan, HA, and synthetically derived polymers [41,42]. Electrospinning, freeze-drying, UV irradiation, and heating are often used as techniques for preparation and production [43]. Atelocollagens, prepared by removing the terminal telopeptides from collagen, have emerged as a popular collagen-based scaffold of choice for addressing foot and ankle pathologies in recent years. In some studies, they have demonstrated superior biocompatibility compared to other collagen-based options [7,44,45].

#### 2.1.4. Gelatin

**Figure 2:**
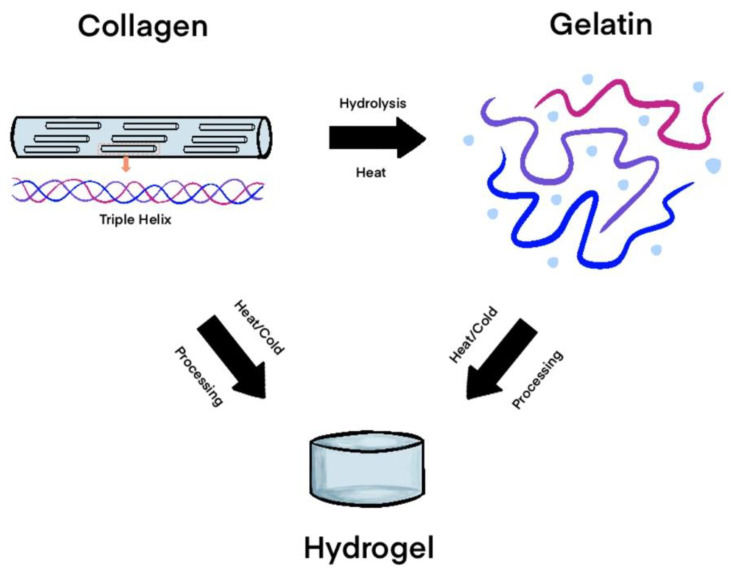
Processing of collagen and gelatin hydrogels.

As a denatured, hydrolyzed form of collagen, gelatin is also an abundant, inexpensive, and common material for gel scaffolds [7,46]. Composed of arginine–glycine–aspartate amino acid sequences, prior studies have demonstrated that gelatin can stimulate osteoclasts and facilitate osteogenesis [46,47]. In its most basic form, gelatin lacks the significant strength and durability required of tissue engineering materials, so it is often modified through cross-linking with agents including glutaraldehyde, carbodiimide, methacrylic anhydride, and diphenylphosphoryl azide [47,48,49,50,51]. A stronger biocompatibility has been obtained by combining gelatin with other natural polymers, such as chitosan [51] (Figure 2).

#### 2.1.5. Hyaluronic Acid

HA is a mammalian glycosaminoglycan composed of repetitions of (β-1,3)-linked N-acetyl-D-glucosamine and (β-1,4)-linked D-glucuronic acid and is abundant in connective tissues such as skin and cartilage [52,53]. At higher molecular weights, HA proves extremely hydrophilic and anti-inflammatory, making it a suitable component for gel scaffolds [7]. The advantages of HA include facilitating cellular interactions with growth factors, mediating cell motility, and regulating osmotic pressure [52]. Multiple methods of creating HA hydrogels have been described, including annealing [54], hydrazide derivate cross-linking [55], transglutaminase cross-linking [56], electrospinning [57], and esterification [58], with transglutaminase cross-linked HA hydrogels showing improved biocompatibility when compared to the other methods [56]. A major disadvantage of HA gel scaffolds is rapid degradation in vivo, which can be partly overcome with certain chemical modification and cross-linking techniques [59]. HA-based hydrogels have been combined with other natural polymers, including chitosan, elastin, and silk, to enhance biocompatibility and function, which has shown promising results in in vitro and animal models but has yet to be evaluated in the context of foot and ankle chondral lesions [60,61,62,63] (Figure 1).

Specific HA scaffolds that have been utilized for foot and ankle surgery include Hyalograft C, produced by Fidia Advanced Biopolymers Laboratories in Abano Terme, Italy. Made from the benzylic ester of HA (HYAFF 11), it consists of a 3D network of non-woven fibers that are 10 to 15 μm thick and varying in size, allowing for optimal cellular contact, growth, and extracellular deposition [64]. First introduced in 1999 for full-thickness cartilage defect repair in the knee [65], it has gained popularity in utilization for ankle chondral lesions in recent years [66,67].

### 2.2. Synthetic

Synthetic polymers offer enhanced mechanical and chemical properties depending on their design by modification. While they have more standardized processing methods and less variability compared to natural scaffolds, concerns about their biocompatibility still exist [7].

#### Polyester-Based Synthetics

Polyester-based polymers have emerged as the most popular and well-studied synthetic polymers over the past few decades for tissue engineering [68,69]. Common polyester biomaterials include polyglycolic acid (PGA), polylactic acid (PLA), combined polylactic–co-glycolic acid (PLGA), polyvinyl alcohol (PVA), polyethylene oxide (PEO), polyethylene glycol (PEG), and polycaprolactone (PCL), although this list is not exhaustive. PGA, PLA, and their copolymer PLGA demonstrate strong biodegradability and are broken down via random hydrolysis into basic components that can be excreted by the body [69,70,71]. However, studies have shown significant inflammatory reactions with larger quantities of these polymers, which have been attributed to their degradation by free radicals [72,73]. Certain preparation techniques, including electrospinning and extraction, have been utilized to enhance the biocompatibility of such polymers, but inflammatory and osteolytic reactions still remain a concern for these materials [74,75]. An example of a widely used PLGA-based scaffold is Bio-Seed (BioTissue Technologies, Freiburg, Germany), a porous construct that is combined with chondrocytes and embedded with fibrin gel [76]. PVA is another hydrophilic, polyester-based polymer and is synthesized via repeated cycles of free-thawing and chemical cross-linking with glutaraldehyde and other chemicals [8].

PEO and PEG are polymers with similar structures and are both currently FDA-approved and widely used for tissue engineering [77]. PEG is a shorter version of the hydrophilic PEO and provokes limited antigenicity and inflammatory reactions in the body [7,78,79]. Both are easily modified to allow for chemical and mechanical adjustments, although they are not sufficient on their own to support cell adhesion and new tissue growth, requiring incorporation with other polyester-based polymers to form adequate hydrogels [7,80,81]. Due to its smaller molecular weight and noninflammatory properties, PEG is often used as a coating adjunct for other scaffolds [82].

**Figure 3:**
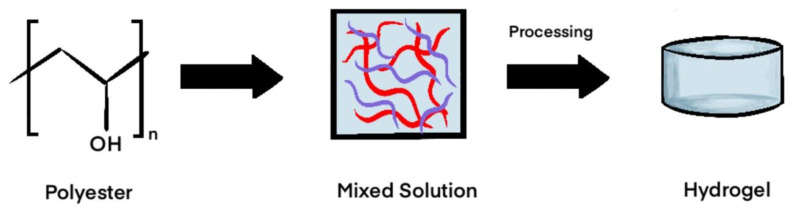
Fabrication of synthetic hydrogels.

PCL is an FDA-approved polyester often used for bone grafting purposes, although there has been an attempt recently to apply it in tissue scaffolding [83]. However, its hydrophobicity and slow degradation compared to other polyesters make it a less suitable option [84,85]. Attempts to combine PCL with more degradable and hydrophilic polymers, including chitosan and other polyesters, have led to improved chemical and mechanical properties, although concerns exist for toxic chemical residuals produced as a byproduct [7,86,87]. Further investigation is required to determine the benefits of PCL applications for scaffolding purposes, especially when alternative suitable polyester options exist (Figure 3).

### 2.3. Hybrids

Hybrid polymers have emerged as viable options for tissue engineering as they can be modified to possess the advantages of both natural and synthetic polymers [88]. Popular options that are currently utilized include gelatin methacrylate (GelMA) and gelatin nanohydroxyapatite (GelnHA). Hybrid systems incorporating nanomaterials have also been developed in recent years.

#### 2.3.1. Gelatin Methacrylate

GelMA is an inexpensive hybrid polymer composed of a gelatin backbone. It is synthesized via the addition of methacrylate groups to this backbone to produce a hydrogel [49]. The gelatin backbone offers advantageous biocompatible properties, enhanced cell adhesion, and adequate degradability compared to true synthetic polymers, while the methacrylation and photo-cross-linking modifications offer the structural integrity that pure natural polymers lack [89,90]. GelMA can be combined with other natural polymers, such as collagen, to facilitate amplified cell proliferation, adhesion, and migration within the scaffold [91].

#### 2.3.2. Gelatin Nanohydroxyapatite

GelnHA is another hybrid hydrophilic polymer utilized for tissue engineering and drug delivery [8]. A combination of gelatin and nano-sized hydroxyapatite (nHA), a synthetic biomaterial with strong protein absorption and adhesion, it can be chemically cross-linked with glutaraldehyde and chloride derivatives or physically cross-linked by repeated freeze–thawing cycles to form hydrogels [8,92,93]. While the gel component provides the architecture to guide chondrocytes into the appropriate 3D structure, the nHA component can stimulate chondrocytes to produce a cartilage matrix in addition to inducing mineralization [94]. Table 1 provides an overview of the principles discussed in this section (Table 1).

#### 2.3.3. Hybrid Systems and Nanomaterials

As hybrid polymers cannot exactly mimic the natural extracellular cell matrix, integrating them with secondary polymer networks can enhance their characteristics and function within the host. Previous attempts to increase hydrogel stiffness come with the cost of stimulating hypertrophic differentiation and reduced permeability [95]. Nanomaterials, such as nanofibers and nanotubes, can improve the mechanical properties of hydrogels, including elasticity, spatial positioning, and stimuli responsiveness [96,97,98,99]. GelMA constructs combined with nanogold, nanosilicates, and nanodiamonds demonstrate increased osteogenesis, stiffness, and decreased inflammatory responses for bone and cartilage gel applications [100,101,102,103].

## 3. Comparison of Gel Types

Overall, there are many advantages to each of the major types of gels. Natural hydrogels possess similar biochemical properties similar to the extracellular matrix, which allows for increased biocompatibility when compared to the other types of gels [104]. Due to their abundance and biocompatibility, the cost of production is relatively low; therefore, they are readily used in many biomedical and research applications [105,106]. However, it is increasingly difficult to store natural hydrogels due to their biodegradability. Additionally, natural gels possess decreased stability when compared to synthetic gels [104,106]. Synthetic gels are increasingly stable and highly tunable but have decreased biocompatibility and tend to instigate inflammatory responses upon implantation [104,107]. The last type of gel is the hybrids, which possess an environmental similarity to natural gels and the stability of synthetic gels [8,107]. Unfortunately, hybrid gels are the most expensive to produce [105].

## 4. Molecular Mechanisms of Gel Interactions

Hydrogels interact with immune cells via multiple mechanisms that depend on the material structures and composition. Softer and more lightly cross-linked hydrogels demonstrate decreased immune reactions, allowing for greater cellular infiltration of the material, while heavily cross-linked hydrogels promote increased inflammation and macrophage recruitment [108]. Hydrogels recruit and activate fibroblasts via RANKL signaling pathways and can control macrophage conversion between pro-inflammatory and anti-inflammatory phases [108,109,110,111]. In biochemical investigations, hyaluronic acid has been proven to polarize primary macrophages to the immunosuppressive phenotype [112]. Hydrogels can also be fabricated to deliver immunomodulatory agents to further facilitate immune reactions, such as vascular endothelial growth factor and cytokines [113,114].

## 5. Mechanobiology

There has been growing emphasis on understanding the interactions between hydrogels and cells, particularly how these networks influence cellular behaviors such as growth, migration, cell–cell communication, and mechano-sensing, which ultimately guide stem cell differentiation and tissue regeneration [109]. Multiple mathematical, statistical, and chemical models have been utilized to characterize the mechanobiology of hydrogels [106]. For example, the elasticity of polymers depends on the force-extension response of fibers, measured through the ratio of persistence length and contour length, with a ratio < 1 indicating higher sensitivity to thermal fluctuations [109]. Osmotic and hydrostatic fluid gradients drive interstitial fluid motion within porous scaffolds, which induces micro-strain distribution and influences the formation of focal adhesions for cell attachment and signaling [115]. Integrin clustering, focal adhesion kinase activation, cytoskeleton rearrangement, cell proliferation, and mesenchymal stem cell differentiation all depend on the local strain distribution [116,117,118].

## 6. Applications of Gels in Foot and Ankle Surgery

The rapid advancements of gels have made them strong candidates in the realm of tissue engineering and regenerative medicine throughout the body [17]. While universally promising, these advancements offer new solutions to augment various orthopedic procedures performed in foot and ankle pathologies [23]. Treatment of osteochondral lesions and osteoarthritis are a few of the pathologies commonly addressed in the literature.

### 6.1. Talar Osteochondral Defects

Osteochondral lesions (OCLs) of the talus commonly occur following acute ankle trauma and can significantly impact the quality of life of active individuals [119,120,121]. While there is no consensus on the most effective treatment strategy, the current literature generally identifies bone marrow stimulation through a microfracture as the primary surgical intervention. However, the outcomes of microfracture procedures can vary, particularly for more extensive or deeper lesions [23,120,121]. This variability has prompted the exploration of various scaffolds designed to enhance the effectiveness of microfracture stimulation.

Natural polymer-based scaffolds, such as HA, have shown considerable promise in treating OCLs of the talus. A study by Yontar evaluated the use of an HA-based cell-free scaffold alongside microfracture for smaller, deeper OCLs in 20 patients. The results indicated a significant improvement in the American Orthopaedic Foot & Ankle Society (AOFAS) scores, which increased from 57.45 to 92.45, while the Visual Analog Scale (VAS) pain scores decreased from 7.05 to 1.65 over an average follow-up period of 20.3 months [122]. Another comparative study investigated arthroscopic treatment with a nanofracture—which uses smaller, deeper channels than a microfracture—alone versus a combination of a nanofracture with an HA scaffold mixed with autologous bone marrow aspirate [123]. The findings revealed that patients receiving the HA scaffold mixed with autologous bone marrow aspirate combined with a nanofracture demonstrated better cartilage quality, higher AOFAS scores, and improved radiological outcomes than the use of a nanofracture alone [123]. The use of HA-based scaffolds has been shown to clinically reduce pain and improve AOFAS scores in the treatment of varying degrees of lesions, indicating their use in the potential augmentation of microfracture stimulation in the treatment of OCLs.

Similar to HA, chitosan has shown comparable efficacy in treating these lesions when evaluated against HA scaffolds. In a study by Akmeşe et al., patients with OCLs of the talus were treated using microfracture techniques alongside either hyaluronan or chitosan-based scaffolds [38]. At 24-month follow-ups, the results demonstrated that patients who received the chitosan scaffold in combination with a microfracture achieved a mean AOFAS score of 82.5, which was similar to the mean AOFAS score of 80.1 for patients treated with hyaluronan. Along with similar AOFAS scores, patients in both groups demonstrated nearly 30% filling of the lesion in question [38].

**Figure 4:**
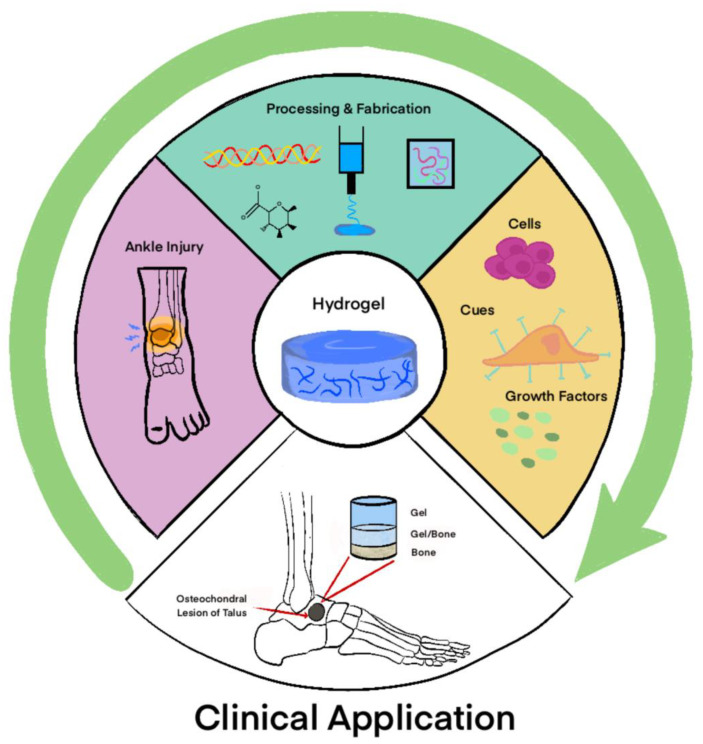
Clinical application of hydrogels for foot and ankle pathologies.

The use of hybrid scaffolds, specifically polyglycolic acid–hyaluronan (PGA-HA) scaffolds, has also shown promising results in single-step arthroscopic treatments for talus OCLs. A study by Kanatli et al. demonstrated that combining a microfracture with a cell-free PGA–HA scaffold was both effective and safe for treating talar OCLs larger than 1.5 cm^2^ [124]. They reported a significant improvement in postoperative AOFAS scores, which increased from an average of 52.8 to 87.1 among 32 patients, with an average follow-up period of 33.8 months. Additionally, MRI assessments indicated that 68.8% of patients showed either complete or hypertrophic filling of the defect [124]. The studies above demonstrate the clinical viability of various gel-based scaffolds in the treatment of talus OCLs; however, more research is needed to determine their efficacy and implementation as standard-of-care for such lesions (Figure 4).

### 6.2. Osteoarthritis Applications

The exploration of hydrogels for the treatment of metatarsal pathologies of the foot is a compelling prospect. In particular, PVA hydrogels have garnered attention as a potential augmentation for the treatment of osteoarthritis (OA) of the first metatarsophalangeal (MTP) joint, or hallux rigidus. However, the literature presents conflicting findings on their efficacy [125,126,127]. While the gold-standard treatment for hallux rigidus is MTP joint arthrodesis, this procedure has been shown to impact gait and restrict footwear selection [128,129].

In an effort to address these problems, motion-sparing procedures using hydrogels have been studied, although with conflicting results. A systematic review found that hemiarthroplasty utilizing PVA implants is associated with significantly higher rates of complications and failures compared to conventional gold-standard treatments, such as arthrodesis [129]. Furthermore, a single-center study has demonstrated that MTP joint arthrodesis provides more effective pain relief than PVA hemiarthroplasty [129]. Conversely, a more recent investigation reported that patients suffering from first MTP joint arthritis who received PVA hydrogel implants via hemiarthroplasty exhibited significant improvements in pain management and hallux dorsiflexion at an average follow-up period of 14.5 months postoperatively [126]. However, these implants demonstrate high rates of implant subsidence and failure, with one study reporting up to 90% of patients experiencing subsidence at final follow-up, in addition to 36% of patients experiencing failure [130]. While these results are promising, the inconsistencies in the literature highlight the need for additional research to better understand the indications and potential risks of failure associated with PVA implants in the treatment of hallux rigidus.

Gel scaffolds have also been studied in the treatment of ankle OA osteoarthritis in patients where conservative retreatment was inadequate [32]. The use of HA–alginate (VersaWrap) was used as an adjunct to ankle arthroscopy in a 56-year-old female. At two months postop, she reported minimal discomfort and full passive and active ranges of motion [32].

While gel utilization for ankle arthritis is still novel, with limited case studies and reviews, scaffold applications for the treatment of knee OA have been investigated and show promising results. At 24 months postoperatively, patients treated for moderate knee OA showed significant improvements in functional outcome and IKDC scores, with only 9.3% of patients experiencing failure requiring revision surgery [131]. In a study of 26 patients with early knee OA, a majority of patients experienced significant improvements in pain and functional outcome scores, with a low complication and failure rate [132]. As applications for OA prove promising in the knee joint, further clinical studies are needed to evaluate their utilization in the ankle.

## 7. Challenges in Scaffold Application

Gel-based scaffolds have seen a substantial rise in their use over the last decade in treating orthopedic foot and ankle pathology and have demonstrated increasing promise for TERM applications. However, considerable challenges remain, greatly limiting the implementation and widespread adoption of this technology. Tissue biocompatibility, structural integrity, and scaffold vascularization are current obstacles that must be addressed to improve the usability and success of these scaffolds.

One of the chief concerns with the implementation of TERM is ensuring biological compatibility with native tissue while minimizing the immune response from the host. As previously mentioned, hydrogels have structural similarities to the native ECM of human tissue, making them desirable for engineering in various tissues [133]. These materials have desirable properties that facilitate favorable cellular responses, therefore increasing biocompatibility within host tissue and minimizing immune response [134]. However, due to their high porosity, hydrogels and other natural scaffolds suffer from poor mechanical strength and may rapidly degrade in vivo [135]. If these scaffold materials degrade too quickly at the implantation site, they may fail to provide the ECM and structural support needed by growing native tissue, therefore limiting regenerative capacity. Recently, the integration of synthetic materials into hydrogel-based and other natural tissue scaffolds has been explored to delay structural degradation in these scaffolds. Studies suggest that the incorporation of bioactive ions and enzymatically degradable linkages may lengthen the support time provided by the scaffold in vivo [136,137,138,139]. Additionally, both natural and synthetic nano-polymers are being incorporated to improve the chemical stability and microenvironment at the tissue site, which has been shown to positively affect cell adhesion, growth, and the extracellular matrix infiltration of cultured cells [140].

Mechanical stability is another key consideration in designing scaffolds for use in locations where the load-carrying capacity is imperative, most notably in orthopedic foot and ankle implants. Many hydrogels on the market have good biocompatibility but lack the mechanical strength required to support the continuous compressive loads experienced by the ankle and foot. Methods to reinforce hydrogels through blending biological and synthetic polymer systems or reinforcement by nanofibers and bioactive ceramics have been developed to achieve increased structural strength [141,142]. While the utilization of synthetic materials can increase structural support and improve the mechanical properties of natural scaffolds, it comes with an increased risk of implant rejection due to the host immune response.

Vascularization is another major challenge to hydrogel scaffold efficacy in regeneration. Proper vascularization is required to supply growth factors, nutrients, and oxygen to tissues during regeneration. However, most hydrogel scaffolds suffer limitations in enabling angiogenesis, causing incomplete regeneration and insufficient integration with native tissues. To overcome this issue, researchers are exploring multiple approaches, such as adding vascular endothelial growth factors (VEGFs), integrating microvascular channels, and using bioactive ceramic biomaterials in scaffolds [133,143,144,145]. Additionally, methods such as ion-loaded hydrogels (such as magnesium- or silicate-based scaffolds) and DNA-based hydrogels with growth factors have been reported to induce vascular ingrowth [146,147,148].

The clinical translation of hydrogels and scaffolds is constrained by practical issues. Preparing hydrogel materials often involves complex steps, such as in situ cross-linking or thermoresponsive gelation, which can make them difficult to handle intraoperatively [149]. Adherent incorporation and proper apposition to host tissues is an enduring challenge in mobile and stress-bearing locations, such as in the foot or ankle. From a clinical perspective, interpatient response variation, non-standardized application methods, and the inability to predict scaffold performance upon implantation have hindered widespread clinical translation. Materials with tunable mechanical and adhesive properties, in addition to research in 3D printing and biofabrication techniques, can help to render these materials appropriate for clinical translation [150,151]. The process of integrating new biomaterials into clinical practice is often lengthy due to strict regulatory guidelines. Achieving the large-scale production of these scaffolds while preserving the material’s biological activity is another key concern. Inconsistencies in raw materials, challenges with sterilization, and stringent quality control standards add to the complexity of manufacturing these materials.

As the usage of gels in foot and ankle surgery is relatively novel, cost analyses of such methods in orthopedics are fairly limited to date. In their recent study, Papadopoulos et al. discussed the fact that gels had the potential to decrease costs associated with knee surgeries [152]. The previous literature has briefly discussed the cost benefits of hydrogel dressings for wound healing, both directly when compared to hydrocolloid dressings and indirectly with regards to reducing healing time [153,154]. While it can be supposed that hydrogel applications in foot and ankle surgery offer similar opportunities to offset costs, further investigation into the cost–benefit analysis of hydrogel applications is required.

The ankle joint proves increasingly challenging for hydrogel applications due to its anatomical structure, high load, and numerous different pathologies [1]. Alternatively, hydrogel applications in the knee joint have been extensively studied and have demonstrated success, potentially due to simpler biomechanics compared to the ankle, allowing for easier facilitation of hydrogel applications [155]. As the application of similar biotechnologies to the ankle joint becomes increasingly popular, it is imperative to investigate and design materials that address its particular mechanical and biochemical requirements [156,157].

## 8. Future Directions

Recent advancements in hydrogel-based scaffold design have introduced bioadaptive materials capable of detecting and responding to environmental stimuli experienced at the implant site. These scaffolds, known as smart hydrogels, can change properties based on physiologic cues, such as biomechanical stress, temperature, or local pH. Therefore, they hold great promise for improving scaffold functionality and durability [158,159]. For instance, the integration of stimuli-responsive porogens can create dynamic macropores within hydrogels, enabling controlled degradation and improved cell infiltration [160]. Moreover, aragonite-based scaffolds are derived from naturally occurring calcium carbonate substances, such as marine corals, and have been most commonly incorporated into hyaluronic acid-based scaffolds in the literature. Early studies utilizing these designs have shown promising results, and many clinician scientists are optimistic about their potential for widespread adoption and implementation, specifically for osteochondral defects [160,161,162,163]. Additional research incorporating graphene oxide or bioactive ceramics in nanocomposite hydrogels has also demonstrated improvements in mechanical and bioactive properties [164,165].

The recent introduction of 3D bioprinting has revolutionized hydrogel-based scaffold manufacturing, allowing for the precise production of patient-specific implants with optimized pore architectures. Extrusion-based, inkjet, and laser-assisted bioprinting enable the deposition of hydrogels with controlled geometries, improving cell adhesion, vascularization, and mechanical properties [166]. However, these printed scaffolds still require improvements in key areas, such as printing quality, biomaterial viscosity, and cell viability [167,168]. A recent development, the free reversible embedding of suspended hydrogels technique, enables printing within a supportive medium, preventing the deformation of soft hydrogels and facilitating layer-by-layer assembly of complex structures [169]. Additionally, hybrid bioceramic–polymer scaffolds have been engineered using digital light-processing bioprinting, enhancing their load-bearing capacity and osteoinductive properties [168]. Researchers are now exploring ways to create pre-vascularized constructs, where endothelial cells and microcapillary networks are embedded within the scaffold before implantation, enabling rapid vascularization for nutrient transport and waste removal at the regeneration site [26]. Further exploration of approaches involving angiogenic ion-incorporated hydrogels, such as magnesium- and silicate-enriched formulations, which have been shown to promote endothelial cell recruitment and vessel formation, is warranted [116,170]. Additionally, recent studies suggest that incorporating dimethyloxalylglycine into hydrogels may activate hypoxia-inducible factors, which are capable of enhancing both bone and vascular tissue formation [171].

## 9. Conclusions

As TERM continues to revolutionize medicine and surgery, there is also growing interest in applying these advancements to foot and ankle surgery. With multiple compelling scaffold prospects across the numerous natural, synthetic, and hybrid polymers currently utilized in TERM, promising results have been described in the treatment of osteoarthritis and osteochondral lesions. However, concerns with material biocompatibility, structural integrity, feasibility during surgery, and degradation still exist and limit the extent of utilization. As researchers continue to develop enhanced polymers and formulations that address current issues, there are many opportunities to increase applications across foot and ankle surgery.

## Figures and Tables

**Table 1:** Main Types of Gels Scaffolds and Templates.

Type	Advantages	Disadvantages	Examples	
Natural	Strong Biocompatibility	Processing Difficulties	Alginate, Chitosan, Collagen, Gelatin, Hyaluronic Acid	[8,29,31,42,62]
Synthetic	Standardized Processing, Limited Variability	Biocompatibility Issues	PGA, PLA, PLGA, PVA, PEO, PEG, PCL	[7,73,74,84,85,86]
Hybrid	Strong Mechanical and Chemical Properties	Minimal	GelMA, GelnHA	[50,93,95,96]

## Data Availability

No new data were created or analyzed in this study.

## Abbreviations

The following abbreviations are used in this manuscript:

TERM	Tissue Engineering and Regenerative Medicine
ECM	Extracellular Matrix
HA	Hyaluronic Acid
PGA	polyglycolic acid
PLA	polylactic acid
PLGA	polylactic-co-glycolic acid
PVA	polyvinyl alcohol
PEO	polyethylene oxide
PEG	polyethylene glycol
PCL	Polycaprolactone
GelMA	gelatin methacrylate
GelnHA	gelatin nanohydroxyapatite
nHA	nano-sized hydroxyapatite
OCL	Osteochondral lesions
AOFAS	American Orthopaedic Foot & Ankle Society
VAS	Visual Analog Scale
PGA-HA	polyglycolic acid–hyaluronan
MTP	metatarsophalangeal
VEGF	vascular endothelial growth factor
